# Dramatic mitigation of bone pain after phosphorus replacement therapy in a subject with FGF23-related hypophosphatemic osteomalacia

**DOI:** 10.1186/s40064-016-3602-6

**Published:** 2016-11-02

**Authors:** Fuminori Tatsumi, Megumi Horiya, Akihito Tanabe, Momoyo Nishioka, Yoshiro Fushimi, Junpei Sanada, Yurie Hirata, Shintaro Irie, Tomoe Kinoshita, Shinji Kamei, Masashi Shimoda, Tomoatsu Mune, Kohei Kaku, Hideaki Kaneto

**Affiliations:** Department of Diabetes, Endocrinology and Metabolism, Kawasaki Medical School, 577 Matsushima, Kurashiki, 701-0192 Japan

**Keywords:** FGF23, Phosphorus, Hypophosphatemic osteomalacia, Bone pain, Fragility fracture, Phosphorus replacement

## Abstract

**Introduction:**

Fibroblast growth factor 23 (FGF23) is secreted from bone and suppresses the absorption of phosphorus in renal proximal tubule and in intestinal tract. Therefore, the increase of serum FGF23 levels leads to hypophosphatemic situations. Tumor-induced osteomalacia is often induced by various tumors, but it is often difficult to identify the localization of tumor, because most of the FGF23-producing tumors are small and could be observed in any part of the body.

**Case description:**

Here we report a case of elderly female subject with FGF23-related hypophosphatemic osteomalacia who repeatedly experienced severe bone pain and fragility fracture in various parts of the body. Although we failed to identify the localization of tumor in this subject even with various examination, after starting phosphorus replacement therapy with relatively small amounts of calcium phosphate (1.5 g/day) (phosphorus content: 270 mg), hypophosphatemia was ameliorated and repeated bone pain was dramatically mitigated without any surgical operation.

**Discussion and Evaluation:**

Even when we fail to identify the localization of tumor in subjects with FGF23-related hypophosphatemic osteomalacia, phosphorus replacement therapy for hypophosphatemia could reduce the bone pain.

**Conclusions:**

We should be aware of the possibility that phosphorus replacement therapy exert marked beneficial effects for the reduction of bone pain in subjects with FGF23-related hypophosphatemic osteomalacia even when we fail to identify tumor localization.

## Background

It is known that fibroblast growth factor 23 (FGF23) is secreted from bone and suppresses the absorption of phosphorus in renal proximal tubule and in intestinal tract. Therefore, the increase of serum FGF23 levels leads to hypophosphatemic situations (Bergwitz et al. 2011; Jonsson et al. 2003; Yu and White 2005). Indeed, FGF23 is an etiology factor of autosomal dominant hypophosphatemic rickets (ADHR) and tumor-induced osteomalacia (Chong et al. 2011, 2013). Tumor-induced osteomalacia is often induced by various tumors such as phosphaturic mesenchymal tumor and ovarian cancer. It is known that FDG-PET/CT and measurement of FGF23 levels in limb with selective venous sampling are useful to estimate the tumor localization and that, if possible, the resection of tumor is the best therapy for the disease. On the other hand, it is often difficult to identify the localization of tumor, since most of the FGF23-producing tumors are benign and small, and these tumors have potential to occur anywhere in the body. Here we report a case of elderly female subject with FGF23-related hypophosphatemic osteomalacia who often experienced severe bone pain and fragility fracture in various parts of the body. Since we failed to identify the localization of tumor in this subject even after various kinds of evaluation, we started phosphorus replacement therapy for the treatment of hypophosphatemia. As the results, repeated bone pain in this subject was dramatically mitigated after the amelioration of hypophosphatemia by phosphorus replacement therapy.

## Case presentation

In 2014, a 75-year-old female was hospitalized in our institution for the careful examination of whole body bone pain and fragility fracture. In 2012, she experienced fracture in proximal end of left tibia and left foot joint. At that time, bone density was as follows: lumbar spine YAM, 79%; left femur YAM, 58%. She was diagnosed as osteoporosis and started taking PTH formulation in local clinic. Furthermore, in 2013, she experienced femur fracture on both sides and multiple rib fracture. At that time, bone density was as follows: lumbar spine YAM, 66%; left femur YAM, 59%. After then, she started taking bisphosphonates. In spite of such therapy, in 2014, she experienced fracture in various parts of the body such as left pubis and left olecranon. After then, she started taking active vitamin D preparations. Finally, she was hospitalized in our institution for the careful examination of such frequent fracture in various parts of body.

**Fig. 1:**
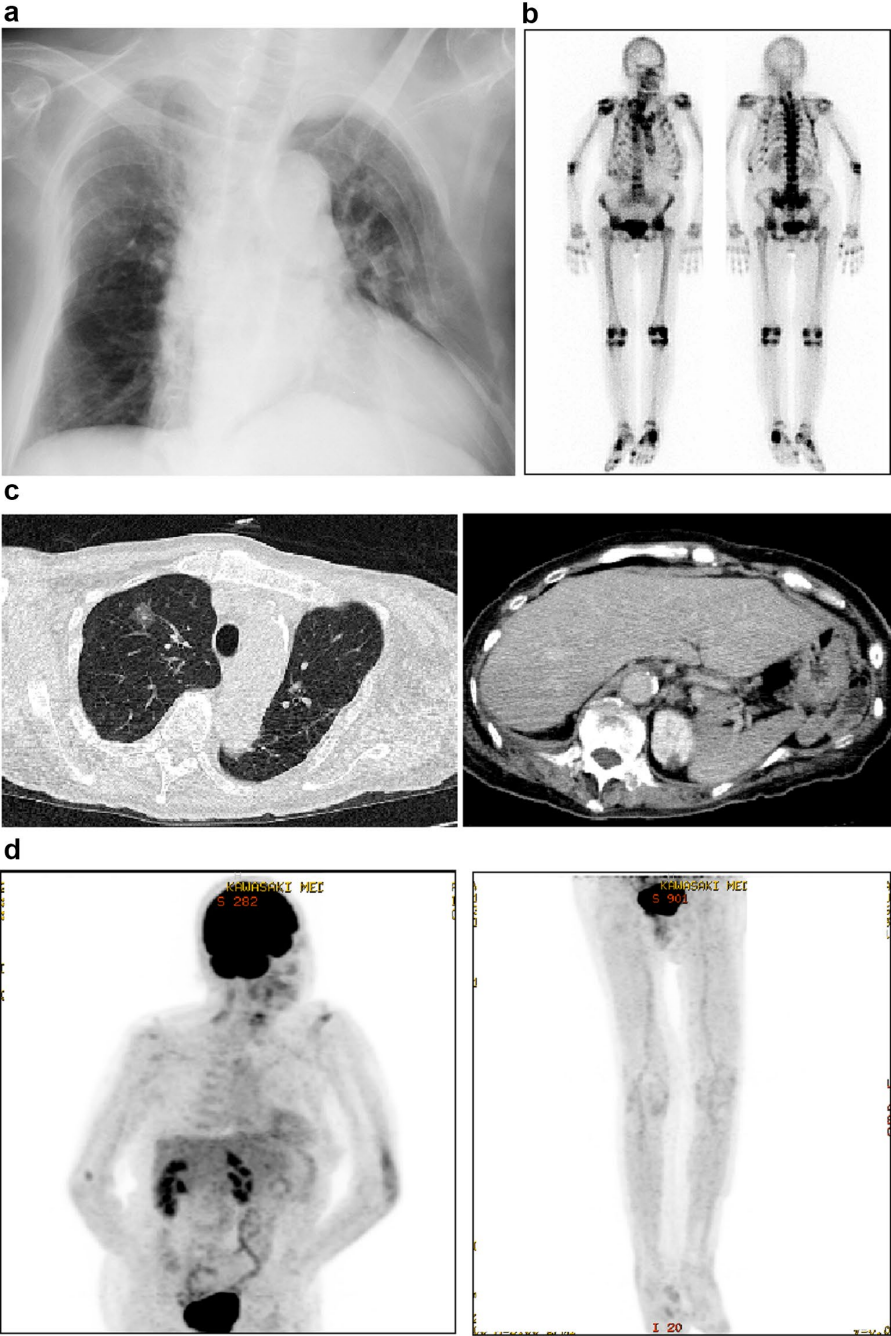
**a** Chest X-ray. There are remnants of multiple rib fracture, marked deformation of the thorax, scoliosis and cardiomegaly. **b** Bone scintigraphy. Abnormal accumulation is observed in ribs on both sides, limb joints. These are the findings after multiple fractures in the body. **c** Computer tomography (CT). There is sciatic fracture on both sides, multiple rib fracture, vertebral compression fracture and marked deformation in the thorax. Neoplastic lesion is not observed in bone and soft tissues. There is localized frosted glass shade in upper right lung field. **d** FDG-PET/CT. There is no abnormal accumulation in the whole body, although slight accumulation is observed in thoracolumbar spine and upper limb joint probably due to arthritis

On admission (August, 2014), her height was 142.5 cm and body weight was 44.7 kg (body mass index: 22.0 kg/m^2^). Although her previous height was unknown, it seemed that her height became shorter apparently due to scoliosis and vertebral compression fracture. Blood pressure was 132/84 mmHg and heart rate was 80 bpm. Body temperature was 36.9 °C. Her consciousness was clear, but she was under bedrest state all day due to severe bone pain throughout the whole body and she had a lot of difficulties even when she tried to turn over in bed. Although there was thoracic deformity, respiratory and heart sound was normal. In addition, there was no abnormality in abdomen. Table 1 shows the clinical characteristics of this subject on admission. Hypophosphatemia (1.5 mg/dL) and hypocalcemia (8.1 mg/dL) (8.3 mg/dL after correction with serum albumin level) were observed. ALP level was high (855 U/L), and BAP (bone-type ALP) level was as high as 88.2 μg/L (normal range 3.8–22.6 μg/L). Serum FGF23 level was also as high as 275 pg/mL (normal range: 10–50 pg/mL). As shown in Table 1, various tumor marker levels all of which we measured were within normal range except for the slight increase of SCC level. As shown in Fig. 1a, in chest X-ray, there were remnants of multiple rib fracture, marked deformation of the thorax, scoliosis and cardiomegaly. In bone scintigraphy, abnormal accumulation was observed in ribs on both sides and limb joints (Fig. 1b). These are the findings after multiple fractures in the body. In computer tomography (CT), there was sciatic fracture on both sides, multiple rib fracture, vertebral compression fracture and marked deformation in the thorax (Fig. 1c). Neoplastic lesion was not observed in bone and soft tissues. There was localized frosted glass shade in upper right lung field. In FDG-PET/CT, there was no abnormal accumulation in the whole body, although slight accumulation was observed in thoracolumbar spine and upper limb joint which was presumably due to arthritis (Fig. 1d). Furthermore, we performed selective venous sampling and measured serum FGF23 levels in various parts of limb, but there was no marked difference in FGF23 levels among various parts of limb as follows: elbow vein, 331 pg/mL (left), 326 pg/mL (right), femoral vein, 299 pg/mL (left), 295 pg/mL (right).

**Table 1:** Clinical characteristics of this subject on admission

WBC	5350/μL	CK	38 U/L	Whole PTH	25.6 pg/mL
Neu	66.6%	CRP	0.08 mg/dL	PTHrP	≤1.0 pmol/L
Lym	25.9%	PBS	83 mg/dL	1,25(OH)_2_D_3_	38.9 pg/mL
Mon	5.1%	HbA1c	5.3%	25(OH)D	7 ng/mL
Eo	2.2%	LDL-chol	95 mg/dL	ACTH	27.8 pg/mL
Bas	0.2%	HDL-chol	41 mg/dL	Cortisol	6.6 μg/dL
RBC	405 × 10^4^/μL	TG	118 mg/dL	DHEA-S	74 μg/dL
Hb	12.8/μL	TSH	0.66 μU/mL	FGF23	275 pg/mL
Ht	37.7/μL	FT4	1.05 ng/dL	Osteocalcin	7.6 ng/mL
plt	18.5 × 10^4^/μL	IgG	931 mg/dL	Serum NTx	22.8 nmol BCE/L
TP	6.1 g/dL	IgA	141.6 mg/dL	TRACP-5b	357 mU/dL
Alb	3.8 g/dL	IgM	48.7 mg/dL	BAP	88.2 μg/L
T-bil	0.3 mg/dL	Na	141 mEq/L	ucOC	1.93 ng/mL
AST	22 U/L	K	3.9 mEq/L	Intact PINP	96.5 μg/L
ALT	17 U/L	IP	1.5 mg/dL	CEA	1.5 ng/mL
γGTP	50 U/L	Ca	8.1 mg/dL	CA19-9	7.9 U/mL
ALP	855 U/L	Corrected Ca	8.3 mg/dL	CA125	8 U/mL
LDH	166 U/L	Urinary Cre	21 mg/dL	SLX	20.0 U/mL
ChE	295 U/L	Urinary P	5.9 mg/dL	AFP	2.4 ng/mL
Crn	0.37 mg/dL	Urinary Ca	9.9 mg/dL	SCC	1.9 ng/mL
BUN	11 mg/dL	FECa	4.46%	CYFRA	1.8 ng/mL
UA	4.8 mg/dL	Tmp/GFR	1.40 mg/dL	ProGRP	45.6 pg/mL

Since we failed to identify the localization of tumor in this subject even with various kind of measurements, we had no choice but to depend on drug therapy and started phosphorus replacement therapy for hypophosphatemia. On September, 2014, we started using calcium phosphate for the treatment of hypophosphatemia in addition to active vitamin D preparations. We used relatively small amounts of calcium phosphate (1.5 g/day) (phosphorus content: 270 mg) in order to avoid the appearance of some side effects in this elderly subject. Such treatment, however, exerted marked beneficial effects in this subject. Hypophosphatemia was significantly ameliorated from 1.5 to 2.5 mg/dL. After discharge from our institution, serum phosphorus levels were within normal range and there was no recurrence of hypophosphatemia with the same amounts of calcium phosphate (1.5 g/day). Serum phosphorus level on March and July in 2015 was 2.5 and 2.7 mg/dL, respectively. Serum calcium levels were also within normal range and there was no recurrence of hypocalcemia. Serum calcium level on March and July in 2015 was 9.1 mg/dL (8.9 mg/dL after correction with serum albumin level) and 9.4 mg/dL (9.1 mg/dL after the correction), respectively. ALP level gradually decreased after the discharge, but it did not reach the normal range. ALP levels on March and July in 2015 was 654 U/L and 591 U/L, respectively. Since this patient hesitated to have an examination about bone density after starting calcium phosphate, we failed to obtain the follow-up data about this point after starting the therapy. We think, however, that it would be better to examine the alteration of bone density after starting the therapy in such cases.

About one month after the treatment, bone pain was decreased by half, and about two months later, she did not feel at all any pain at rest. Finally, about three months later, she became able to stand up without any difficulties and perform a light labor. It is noted here there was no special treatment for this subject except for the phosphorus replacement therapy.

Taken together, bone pain was dramatically reduced after phosphorus replacement therapy in an elder female subject with FGF23-related hypophosphatemic osteomalacia.

## Conclusion and discussion

In this report, we showed a case of elder female subject with FGF23-related hypophosphatemic osteomalacia. She experienced severe bone pain and fragility fracture very frequently in various parts of the body, but after starting phosphorus replacement therapy for hypophosphatemia, marked beneficial effects were obtained. After the phosphorus replacement therapy, hypophosphatemia was significantly ameliorated which was accompanied by the dramatic reduction of bone pain.

The absorption of phosphorus in renal proximal tubule is one of the most important determinants for serum phosphorus concentration. FGF23 suppresses such absorption of phosphorus in renal proximal tubule reduces, which leads to reduction of serum phosphorous levels. In addition, FGF23 functions to suppress the absorption of phosphorus in intestinal tract. Such suppression of phosphorus absorption leads to the development of hypophosphatemic diseases such as tumor-induced osteomalacia. In general, the resection of the tumor is the best way for tumor-induced osteomalacia, but in this case we failed to identify the localization of tumor even with various kind of evaluation. Even in such situations, however, phosphorus replacement therapy for hypophosphatemia markedly reduced the bone pain. Therefore, even when we fail to identify tumor localization, we should start phosphorus replacement therapy. In addition, only small amounts of calcium phosphate (1.5 g/day) (phosphorus content: 270 mg) were used for this subject in order to avoid the appearance of side effects, but bone pain which we failed to suppress with any treatment was dramatically mitigated by the phosphorus replacement therapy, and we think that the amelioration of hypophosphatemia with this treatment would lead to the prevention of further fracture.

It is well know that bone pain could be due to bone metastasis of various kinds of malignant diseases. Especially, lung cancer, breast cancer and melanoma often bring out bone metastasis. However, she had no symptoms and no findings in CT and PET-CT with which we should suspect that she had lung cancer. In addition, she had no symptom in breast and there was no specific observation with which we should suspect that she had breast cancer. There was no specific findings in peripheral blood test with which we should suspect that she had some hematological disease, although we did not perform bone puncture. Furthermore, there was no specific findings in her skin in the whole body with which we should suspect that she had some disease such as melanoma.

There is a limitation in this study. Hospitalization period of this subject was relatively short (12 days) (August 12–23, 2014) due to the convenience of this subject. In addition, after the reduction of bone pain, this subject denied to have various examination. Therefore, although we confirmed that the reduction of bone pain was closely associated with the amelioration of hypophosphatemia, we failed to examine whether the reduction of bone pain is associated with various objective indicators such as grip strength and bone density. We think we should have performed such examination so that we could more objectively show the benefit of small amounts of phosphorus replacement therapy.

Taken together, we should be aware of the possibility that relatively small amounts of calcium phosphate would be enough to reduce the symptom of bone pain and possibly lead to the prevention of fragility fracture especially in elderly subjects as observed in this subject. We think that the findings in this report would be useful from the clinical point of view.

## Abbreviations

WBC: white blood cell
Neu: neutrophil
Lymph: lymphocyte
Mono: monocyte
Eos: eosinophil
Baso: basophil
Mono: monocyte
RBC: red blood cell
Hb: hemoglobin
Ht: hematocrit
plt: platelet
TP: total protein
Alb: albumin
T-bil: total bilirubin
AST: aspartate transaminase
ALT: alanine transaminase
ɣ-GTP: gamma-glutamyl transferase
ALP: alkaline phosphatase
LDH: lactate dehydrogenase
ChE: cholinesterase
Cre: creatinine
BUN: blood urea nitrogen
UA: uric acid
CK: creatine kinase
CRP: C-reactive protein
PBS: plasma blood glucose
HbA1c: hemoglobin A1c
LDL chol: low density lipoprotein cholesterol
HDL chol: high density lipoprotein cholesterol
TG: triglyceride
TSH: thyroid-stimulating hormone
FT4: free thyroxine
IgG: immunoglobulin G
IgA: immunoglobulin A
IgM: immunoglobulin M
Na: sodium
K: potassium
Cl: chloride
IP: phosphorus
Ca: calcium
EFCa: fractional excretion of calcium
PTH: parathyroid hormone
PTHrP: parathyroid hormone-related protein-C
1,25(OH)_2_D_3_: 1-25-dihydroxyvitamin D3
25(OH)D: 25-hydrovitamin D
ACTH: adrenocorticotropic hormone
DHEA-S: dehydroepiandrosterone sulfate
FGF23: fibroblast growth factor 23
NTx: type I collagen cross-linked N-telopeptide
TRACP-5b: tartrate-resistant acid phosphatase-5b
BAP: bone-type alkaline phosphatase
ucOC: undercarboxylated osteocalcin
PINP: type I procollagen N-terminal propeptide
CEA: carcinoembryonic antigen
CA19-9: carbohydrate antigen 19–9
CA125: cancer antigen 125
SLX: sialyl lewis x antigen
AFP: alpha-fetoprotein
SCC: squamous cell carcinoma
CYFRA: cytokeratin fragment
ProGRP: pro gastrin-releasing peptide
